# Non-alcoholic fatty liver disease as a risk factor for cholangiocarcinoma: a systematic review and meta-analysis

**DOI:** 10.1186/s12876-017-0696-4

**Published:** 2017-12-08

**Authors:** Nicha Wongjarupong, Buravej Assavapongpaiboon, Paweena Susantitaphong, Wisit Cheungpasitporn, Sombat Treeprasertsuk, Rungsun Rerknimitr, Roongruedee Chaiteerakij

**Affiliations:** 1 Division of Gastroenterology, Department of Medicine, Faculty of Medicine, Chulalongkorn University and King Chulalongkorn Memorial Hospital, 1873 Rama IV Road, Patumwan, Bangkok, 10330 Thailand; 2 Department of Physiology, Faculty of Medicine, Chulalongkorn University and King Chulalongkorn Memorial Hospital, Bangkok, Thailand; 3 Department of Parasitology, Faculty of Medicine, Chulalongkorn University and King Chulalongkorn Memorial Hospital, Bangkok, Thailand; 4 Division of Nephrology, Department of Medicine, Faculty of Medicine, Chulalongkorn University and King Chulalongkorn Memorial Hospital, Bangkok, Thailand; 5 Division of Nephrology and Hypertension, Mayo Clinic, Rochester, MN USA

**Keywords:** Non-alcoholic fatty liver disease, Non-alcoholic steatohepatitis, NAFLD, NASH, Cholangiocarcinoma, Bile duct cancer, Risk factor

## Abstract

**Background:**

Non-alcoholic fatty liver disease (NAFLD) has been recently identified as a risk factor of gastrointestinal tract cancers, especially hepatocellular carcinoma, and colorectal cancer. Whether NAFLD is a risk factor for cholangiocarcinoma (CCA) remains inconclusive. The aim of this study is to determine a potential association between NAFLD and CCA, stratifying by its subtypes; intrahepatic CCA (iCCA), and extrahepatic CCA (eCCA).

**Methods:**

A search was conducted for relevant studies published up to April 2017 using MEDLINE, EMBASE, Scopus and Cochrane databases. Odds ratio (OR) and adjusted OR with 95% confidence interval (CI) were estimated using a random-effects model. Subgroup analyses were conducted with study characteristics.

**Results:**

Seven case-control studies were included in the analysis, with a total of 9,102 CCA patients (5,067 iCCA and 4,035 eCCA) and 129,111 controls. Overall, NAFLD was associated with an increased risk for CCA, with pooled OR of 1.95 (95%CI: 1.36–2.79, *I* ^*2*^=76%). When classified by subtypes, NAFLD was associated with both iCCA and eCCA, with ORs of 2.22 (95%CI: 1.52–3.24, *I* ^*2*^=67%) and 1.55 (95%CI: 1.03–2.33, *I* ^*2*^=69%), respectively. The overall pooled adjusted ORs were 1.97 (95%CI: 1.41–2.75, *I* ^*2*^=71%), 2.09 (95%CI, 1.49–2.91, *I* ^*2*^=42%) and 2.05 (95%CI, 1.59–2.64, *I* ^*2*^=0%) for all CCAs, iCCA, and eCCA, respectively.

**Conclusions:**

This meta-analysis suggests that NAFLD may potentially increase the risk of CCA development. The magnitude of NAFLD on CCA risk is greater for iCCA than eCCA subtype, suggestive of a common pathogenesis of iCCA and hepatocellular carcinoma. Further studies to confirm this association are warranted.

**Trial registration:**

The protocol for this study was registered with PROSPERO (International Prospective Register of Systematic Reviews; no. CRD42016046573).

**Electronic supplementary material:**

The online version of this article (10.1186/s12876-017-0696-4) contains supplementary material, which is available to authorized users.

## Background

Cholangiocarcinoma (CCA) is a cancer arising from bile duct epithelium. The incidence of CCA varies by geographic regions, ranging from 0.4–3.4 per 100,000 persons-year in North America and Europe to 1–85 per 100,000 persons-year in East Asia [1]. Although CCA is a relatively uncommon cancer, its incidence has been rising worldwide over the past decade [1]. It remains unclear why the incidence of CCA has been on the rise. This might be due to an increase in prevalence of some host or environmental factors potentially related to CCA development.

CCA is broadly categorized into 2 subtypes by anatomic locations as intrahepatic (iCCA) and extrahepatic (eCCA) subtypes. The two subtypes hold different genetics, presentations, management, and outcomes [2]. Since diagnosis in early stages of disease is difficult, most patients have poor prognosis. Thus, recognition of CCA risk factors would potentially identify individuals at risk and may consequently improve patient outcomes.

Non-alcoholic fatty liver disease (NAFLD) is a spectrum of liver disease, ranging from fatty liver to non-alcoholic steatohepatitis (NASH) and cirrhosis. It is estimated that one-third of the general population has NAFLD [3–5]. Accumulating evidence suggested that NAFLD was associated with an increased risk of various cancers, including hepatocellular carcinoma (HCC), esophageal, gastric, colorectal, breast, and prostate cancer [6, 7]. Recent data suggested that NAFLD might increase the risk of CCA, particularly the iCCA subtype [8–10]. Whether NAFLD is associated with the other subtype of CCA remains uncertain.

To determine an association between NAFLD and CCA including its subtypes, we conducted a meta-analysis of case-control and cohort studies.

## Methods

### Data sources and searches

The protocol for this study was registered with PROSPERO (International Prospective Register of Systematic Reviews; no. CRD42016046573). We performed a systematic review in accordance with the PRISMA (Preferred Reporting Items for Systematic Reviews and Meta-analyses) guidelines (Additional file 1: Data S1) [11]. We searched several databases including Ovid MEDLINE, Epub Ahead of Print, Ovid Medline In-Process & Other Non-Indexed Citations, Ovid MEDLINE, Ovid Cochrane Central Register of Controlled Trials, Ovid EMBASE and Scopus from the inception of the databases through April 5, 2017. The search strategy was designed and conducted by an experienced librarian with input from the study’s principal investigator. Searching terms included cholangiocarcinoma, NAFLD, NASH, risk factors, and their related terms (detail of the full search strategy is provided in Additional file 2: Data S2A, S2B, and S2C). Search strategies were confined to human studies and case-control, cohort, or trial studies. Title and abstract of included studies were screened. References of the included studies and all articles that cited the included studies were reviewed for other potential relevant studies.

### Study selection

Primarily, two authors (NW and BA) independently screened titles and abstracts of all studies that determined any risk factors for CCA and reached for the full text. From full text, studies were included if they that met all of the following criteria: (i) case-control, cohort or trial study, (ii) NAFLD or NASH, defined by either histopathological examination, imaging study or International Classification of Diseases, Ninth Revision (ICD-9) or ICD-10 codes, as one of the exposure of interests, (iii) CCA either iCCA, eCCA, or both as outcome of interest, (iv) study that provided adequate information for calculation of odds ratio (OR) or relative risk for case-control study and cohort study, respectively. Studies of patient cohorts with recurrent CCA or combined hepatocellular-cholangiocarcinoma were excluded. If the same patient cohort was included in more than one study, the study with a larger sample size and a higher quality score assessed by Newcastle-Ottawa scale (NOS) scale was included.

### Data extraction and quality assessment

Data were independently extracted from full-text articles by two authors (NW and BA). Disagreements were identified and discussed with the third author (RC). The information extracted included diagnostic criteria of NAFLD/NASH, a crude number of individuals with and without NAFLD/NASH in the case and control groups, the source of controls (population-based or hospital-based), number of participants, country where a study was conducted, and publication year. Adjusted odds ratio (AOR) and covariates in the adjusted analysis model were also extracted.

The quality of studies was evaluated using Newcastle-Ottawa scale (NOS) [12], which comprises three sections: selection (up to 4 points), comparability (up to 2 points) and outcome (up to 3 points), with a maximum of 9 points. The study quality was classified as poor (score 0–3), fair (score 4–6), or good (score 7–9) [13].

### Data synthesis and analysis

Pooled OR along with 95% confidence interval (CI) of CCA, iCCA, and eCCA were estimated from the crude number of study patients using a random-effects model. Heterogeneity among studies was assessed using both the *I* ^2^ statistics and *P* value. An *I* ^2^ value of >50% indicates substantial heterogeneity. Univariate random-effects model meta-regressions of the adjusted OR (AOR) from the included studies were performed. Publication bias was assessed by the Egger test. All analyses were performed using Comprehensive Meta-Analysis Software version 2.0, and the Metan and Metareg Commands of Stata 11 (College Station, TX).

## Results

### Literature search

**Fig. 1:**
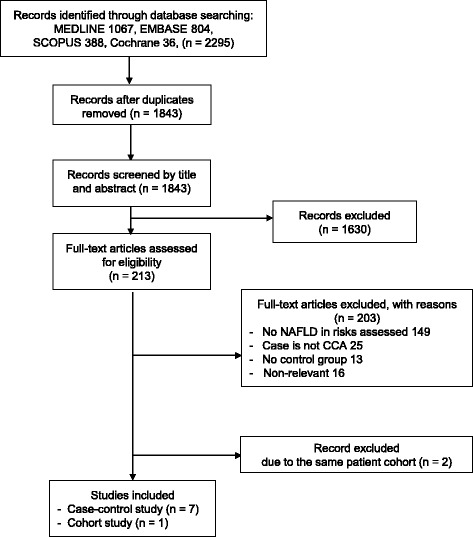
Flow diagram of search methodology and selection process

After excluding duplicates, 1843 articles that met the search criteria were identified. After title and abstract screening, 213 studies that met the selective criteria were further assessed (Fig. 1). Two hundred and three studies were excluded due to the following reasons: NAFLD was not assessed in the study (*n* = 149); the case group was not diagnosed with CCA (*n* = 25); no control group in the study (*n* = 13); and irrelevant study (*n* = 16). There were ten potential relevant studies, of which studies from Chaiteerakij et al. [14] and Haung et al. [15] were excluded because their patient cohort was a part of the cohort in studies of Choi et al. [16] and Chang et al. [17], respectively (detail in Additional file 2: Data S3). Finally, eight studies met the criteria: seven case-control studies and one cohort study. All studies were published in full-text article.

Given the difference in study design, 7 case-control studies were included in this meta-analysis (Table 1) [9, 10, 16–20]. The single cohort study was analyzed separately because this study provided limited information, i.e. CCA was not classified as iCCA or eCCA; and performed restricted analysis, i.e. the analysis was not adjusted for potential confounders (Additional file 2: Data S4) [21].

**Table 1:** Characteristics of 7 included case-control studies

Author, year	Country	Dates	NAFLD Diagnosis	Source	Study quality^a^
Welzel et al. 2007 [10]	US	1999–2009	ICD9	Community	Fair
Zhou et al. 2009 [20]	China	2003–2006	Imaging	Hospital	Good
Chang et al. 2013 [17]	Taiwan	2004–2008	ICD9	Community	Fair
Lee et al. 2015 [18]	South Korea	2007–2013	Histology or imaging	Hospital	Good
Kinoshita et al. 2016 [9]	Japan	1995–2014	Histology	Hospital	Fair
Choi et al. 2016 [16]	US	2000–2014	Histology or imaging	Hospital	Good
Stepien et al. 2016 [19]	10 European countries	1992–2000	Hepatic steatosis index	Community	Good

*NAFLD* Non-alcoholic fatty liver disease

^a^Based on Newcastle-Ottawa Scale (NOS). See Additional file 2: Data S5

### Study characteristics

In the 7 case-control studies, the period of participant enrollment ranged from 1992 to 2014. There was a total of 9102 CCA patients (5067 iCCA and 4035 eCCA) and 129,111 controls. Overall, 426 (4.7%) CCA cases and 1018 (0.8%) controls had underlying NAFLD or NASH (Table 2). Three studies were conducted in Western countries [10, 16, 19] of which approximately 97% of participants were white. Other 4 studies were conducted in Asian countries with no demonstrated proportion of race [9, 17, 18, 20]. Three studies were community-based case-control studies [10, 17, 19], while the other 4 studies were hospital-based case-control studies [9, 16, 18, 20]. NAFLD was diagnosed by histopathology in 1 study [9], by radiologic imaging in 1 study [20], and by either histopathology or imaging in 2 studies [16, 18]. In the other 2 studies, NAFLD was diagnosed by ICD-9 codes: 571.8 [10, 17]. Only one study used hepatic steatosis index for diagnosis of NAFLD [19]. Among the 7 included studies, 4 studies clearly stated that all participants in control groups underwent the same procedure as cases for diagnosis of NAFLD, such as imaging, histopathology examination, or laboratory test for hepatic steatosis index [9, 18–20]. Regarding the study quality, four studies were classified as good quality [16, 18–20] and the other 3 studies were classified as fair quality [9, 10, 17] (Table 1 and Additional file 2: Data S5).

**Table 2:** Number of patients with NAFLD and cholangiocarcinoma in the included case-control studies

Author, year	Number of CCA cases (n)						Number of controls (n)	
	iCCA		eCCA		All			
	Total	With NAFLD	Total	With NAFLD	Total	With NAFLD	Total	With NAFLD
Welzel et al. 2007 [10]	535	5 (0.9%)	549	4 (7.3%)	1084	9 (0.8%)	102,782	353 (0.34%)
Zhou et al. 2009 [20]	317	6 (1.9%)	–	–	317	6 (1.9%)	634	8 (1.2%)
Chang et al. 2013 [17]	2978	156 (5.2%)	2179	89 (4.1%)	5157	245 (4.8%)	20628^a^	410 (2.0%)
Lee et al. 2015 [18]	–	–	81	17 (21.0%)	81	17 (21.0%)	162	28 (17.3%)
Kinoshita et al. 2016 [9]	34	15 (44.1%)	–	–	34	15 (44.1%)	69	13 (18.8%)
Choi et al. 2016 [16]	1169	61 (5.2%)	1226^b^	52 (4.2%)	2395	113 (4.7%)	4769	181 (3.8%)
Stepien et al. 2016 [19]	34	21 (61.8%)	–	–	34	21 (61.8%)	67	25 (37.3%)

*eCCA* Extrahepatic cholangiocarcinoma, *iCCA* Intrahepatic cholangiocarcinoma, *NAFLD* Non-alcoholic fatty liver disease

^a^Comprised 11,912 controls for iCCA cases, of whom 236 had NAFLD; and 8716 controls for eCCA cases, of whom 174 had NAFLD

^b^Included 231 patients with distal CCA, of whom 15 had NAFLD; and 995 patients with perihilar CCA, of whom 37 had NAFLD

### NAFLD and risk of CCA

**Fig. 2:**
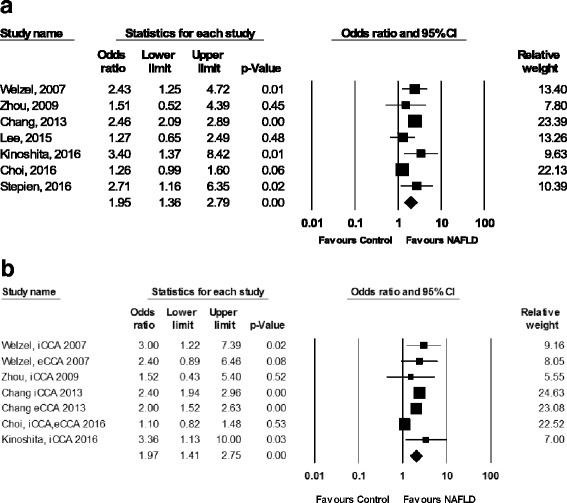
Forest plots of seven studies showing unadjusted odds ratio (**a**) and adjusted odds ratio (**b**) of the association between NAFLD and the risk of cholangiocarcinoma

Of the 7 studies included, four studies found a statistically significant positive association between NAFLD and CCA [9, 10, 17, 19], while the other three studies did not find such association (Fig. 2a) [16, 18, 20]. Overall, pooled OR of NAFLD were 1.95 (95% CI: 1.36–2.79) for CCA risk, with statistically significant heterogeneity among studies (*I* ^*2*^ = 76%, *P* < 0.01, Table 3).

**Table 3:** Stratified analyses of unadjusted odds ratio of CCA by study characteristics

Groups	# of studies	Studies	Number of patients				Pooled OR (95% CI)	Heterogeneity	
			Case		Control			*P* value	*I* ^*2*^ (%)
			Total	With NAFLD	Total	With NAFLD			
All CCAs	7	[9, 10, 16–20]	9102	426	129,111	1018	1.95 (1.36–2.79)	<0.01	76
CCA subtype									
iCCA	6	[9, 10, 16, 17, 19, 20]	5067	264	120,233	816	2.22 (1.52–3.24)	0.01	67
eCCA	4	[10, 16–18]	4035	162	116,429	736	1.55 (1.03–2.33)	0.02	69
Study region									
Asian	4	[9, 17, 18, 20]	5589	283	21,493	459	2.13 (1.47–3.10)	0.19	37
Western	3	[10, 16, 19]	3513	143	107,618	559	1.83 (1.05–3.18)	0.06	65
Study quality									
Good	4	[16, 18–20]	2827	157	5632	242	1.33 (1.07–1.65)	0.39	0
Fair	3	[9, 10, 17]	6275	269	123,479	776	2.48 (2.13–2.90)	0.79	0

*CCA* Cholangiocarcinoma, *eCCA* Extrahepatic cholangiocarcinoma, *iCCA* Intrahepatic cholangiocarcinoma, *OR* Odds ratio

Subgroup analyses by study region and quality of the study were performed. The pooled ORs for CCA were 1.83 (95% CI: 1.05–3.18, *I* ^*2*^ = 65%; *P* = 0.06), and 2.13 (95% CI: 1.47–3.10, *I* ^*2*^ = 37%; *P* = 0.19) for studies conducted in Western and Asian countries, respectively. The good quality studies had pooled OR of 1.33 (95% CI: 1.07–1.65, *I* ^*2*^ = 0%; *P* = 0.39) and the fair quality studies had pooled OR of 2.48 (95% CI: 2.13–2.90, *I* ^*2*^ = 0%; *P* = 0.79) (Table 3).

### NAFLD and risk of iCCA and eCCA subtype

Of the six studies presenting results of NAFLD and iCCA risk [9, 10, 16, 17, 19, 20], four studies found a significant association between NAFLD and iCCA risk [9, 10, 17, 19]. The pooled OR of NAFLD were 2.22 (95% CI: 1.52–3.24, *I* ^*2*^ = 67%; *P* = 0.01) for iCCA risk.

There were four studies presenting results of NAFLD and risk of eCCA [10, 16–18]. Only one study found that NAFLD was significantly associated with eCCA [17]. However, when combining all 4 studies, statistical significant association between NAFLD and eCCA was detected, with pooled OR of 1.55 (95% CI: 1.03–2.33, *I* ^*2*^ = 69%; *P* = 0.02).

### Pooled adjusted odds ratios of NAFLD and CCA risk

All but two studies provided AOR of NAFLD for CCA risk (Additional file 2: Data S6). The Lee et al. study [18] did not present AOR because NAFLD was not significantly associated with CCA in the univariate analysis, and thus was not included in the multivariate analysis.The Stepein et al. study [19] did not show multivariate analysis model. The pooled AORs for CCA were 1.97 (95% CI: 1.41–2.75, *I* ^*2*^ = 71%; *P* < 0.001) [9, 10, 16, 17, 20]. The pooled AORs for iCCA and eCCA were 1.98 (95%CI: 1.26–2.69, *I* ^*2*^ = 47%; *P* = 0.11) [9, 10, 16, 17, 20] and 2.05 (95%CI: 1.59–2.64, *I* ^*2*^ = 0%; *P* = 0.90) [10, 16, 17], respectively (Fig. 2b).

### Publication bias

No publication bias was detected by the Egger’s regression asymmetry test, with *P* = 0.82 and 0.86 for unadjusted and adjusted OR of NAFLD, respectively. However, due to the limited number of the included studies, the interpretation of Egger’s test should be done cautiously.

## Discussion

The present meta-analysis of seven case-control studies found that NAFLD was significantly associated with an increased risk of CCA for both iCCA and eCCA subtypes. This finding might in part explain the recent trend of rising incidence of CCA.

Although the mechanism by which NAFLD causes CCA development has not yet been well studied, it is biologically plausible that NAFLD promotes cholangiocarcinogenesis directly through an induction of hepatic inflammation, or indirectly via cirrhosis. Cirrhosis, regardless of etiology, has recently been recognized as a risk factor for CCA [10, 16, 17]. Alteration of the microenvironment in presence of cirrhosis is a hallmark of carcinogenesis [22]. Up to 5% of patients with NAFLD developed cirrhosis during 8 years of follow-up [23]. In vivo and in vitro studies found that a number of signaling molecules crucial for carcinogenesis were aberrantly expressed in NAFLD [24, 25]. These include an increased expression of pro-inflammatory cytokines, particularly interleukin-6 (IL-6) [24], which plays a pivotal role in induction of cholangiocyte proliferation [26]. Tumor necrosis factor-alpha (TNF-α), another pro-inflammatory cytokine, activated inducible nitric oxide synthase (iNOS) leading to nitric oxide production. This change consequently promotes DNA damage and inhibits DNA repair mechanisms [27]. iNOS activation also upregulated COX2 expression, which promotes cholangiocyte growth [28].

It is important to note that the prevalence of NAFLD in this meta-analysis varied from 0.8 to 44.1% and 0.3 to 18.8% in CCA cases and controls, respectively. These numbers were relatively low as compared to previous studies reporting prevalence of NAFLD in the general population of 11–45% and 8–42% in North America and Asia, respectively [3, 4]. Substantial variation among studies can be explained by differences in diagnostic methods of NAFLD, with the highest prevalence in studies that NAFLD was diagnosed by histopathological exam (15.6–69.3%), followed by imaging (20.0–30.9%), and elevation of liver enzyme (7.9–14.9%) [5]. The study from Stepien et al. [19] which used hepatic steatosis index to diagnosed NAFLD demonstrated the highest prevalence of NAFLD of 61.8% and 37.3% in the case and control groups, respectively. Moreover, the included studies that used histopathological exam as part of NAFLD diagnosis [9, 16, 18] had significantly higher prevalence than the studies that used only imaging or ICD coding [10, 17, 20] (4.7–44.1% vs. 0.8–4.5%). This could reflect an underestimated number of patients with NAFLD in the studies that used only imaging as diagnostic criteria.

Our finding showed the effect of NAFLD was more pronounced for the development of iCCA than eCCA (pooled OR of 2.22 vs. 1.55). This finding is consistent with the previous study showing that iCCA was more associated with chronic liver diseases than eCCA [1]. Moreover, a recent in vivo study showed that iCCA was originated from hepatic cell lineage rather than bile duct cell lineage [29]. On the other hand, eCCA, including perihilar and distal CCA, tended to be associated with biliary tract diseases, as supported by a strong association between primary sclerosing cholangitis and perihilar CCA [1].

As expected, the estimated pooled OR of NAFLD obtained from community-based studies was greater than that obtained from hospital-based studies (2.46 vs. 1.47) (Data not shown). This can be explained by a relatively lesser proportion of controls diagnosed with NAFLD in community-based studies compared to hospital-based studies. Because individuals with NAFLD are mostly asymptomatic, thus controls in community-based studies might have less likelihood to undergo radiologic images, which could potentially increase the possibility of under-detection of NAFLD in controls in community-based studies. This is suggested by the finding that the proportion of NAFLD in controls in community-based studies were lower than the previous reports [3–5]. To minimize the impact of under-detection rate of NAFLD in the control groups, we performed subgroup analysis of the studies that diagnostic procedures, i.e. laboratory test, radiologic imaging, or histopathological assessment, were performed in all cases and controls and found that the estimated pooled OR was 2.0. We believe that this pooled OR was the estimate most approximate to the true value.

The present meta-analysis has some limitations. Diagnostic methods for NAFLD were different among studies. This bias was accounted by the aforementioned analysis of subgroup by the same diagnostic methods for both case and control groups. The results of the two analyses were consistent, implying that our finding was not influenced by NAFLD diagnosis method. In addition, subgroup analyses of some underlying diseases predisposing to CCA, e.g. primary sclerosing cholangitis, cholelithiasis, biliary tract infection, and other chronic liver diseases, cannot be performed due to the limited number of participants with these conditions. Because CCA is considered to be a relatively rare cancer, the number of publications in this field is quite small compared to other cancers. Consequently, the number of studies qualified for this meta-analysis was limited. Accordingly, the probability of type I errors in the process of hypothesis testing existed, particularly with multiple subgroup analyses. However, the subgroup analyses are necessary to explore the finding of the main analysis. We limited the subgroup analyses to the three most important subgroups, including CCA subtypes, study region, and study quality. Another limitation was that NAFLD is a spectrum of disease, ranging from simple steatosis to cirrhosis; in this study, we were not able to estimate the magnitude of impact of steatosis, steatohepatitis or NASH-related cirrhosis on CCA risk individually because they used different diagnostic methods and most papers grouped steatosis and steatohepatitis together. Lastly, cirrhosis itself has recently been recognized as a risk factor for CCA, the possibility that the observed association between NAFLD and CCA in this study was confounded by cirrhosis cannot be excluded. A study to determine whether the increased risk of CCA in patients with NAFLD is independent to cirrhosis status is needed.

## Conclusions

This meta-analysis suggests that NAFLD may potentially increase the risk of CCA development. The magnitude of NAFLD on CCA risk is greater for iCCA than eCCA subtype, suggestive of a common pathogenesis of iCCA and hepatocellular carcinoma. Further studies to elucidate both the strength of the association between NAFLD and CCA, as well as the mechanisms that underlie this relationship are warranted.

## Additional files

Additional file 1: Data S1. PRISMA 2009 checklist for systematic review. (DOC 66 kb)

Additional file 2: Data S2. A. Search strategy for Ovid MEDLINE Epub Ahead of Print, In-Process & Other Non-Indexed Citations, Ovid MEDLINE Daily and Ovid MEDLINE. **Data S2** B. Search strategy for EMBASE. **Data S2** C. Search strategy for Scopus. **Data S3** Exclusion of studies that had the same patient cohort. **Data S4** Data of the included cohort study. **Data S5** Quality assessment of included paper by Newcastle-Ottawa scale (NOS). **Data S6** Adjusted odds ratios shown in the included studies. (DOCX 41 kb)

## Abbreviations

AOR: Adjusted odds ratio
CCA: Cholangiocarcinoma
CI: Confidence interval
eCCA: Extrahepatic cholangiocarcinoma
HCC: Hepatocellular carcinoma
iCC: Intrahepatic cholangiocarcinoma
NAFL: Non-alcoholic fatty liver disease
NASH: Non-alcoholic steatohepatitis
OR: Odds ratio
