# Supplementary material for: Non-alcoholic fatty liver disease as a risk factor for cholangiocarcinoma: a systematic review and meta-analysis
Source: BMC Gastroenterol. 2017 Dec 8;17:149. doi: 10.1186/s12876-017-0696-4 (PMC5721586; doi:10.1186/s12876-017-0696-4)
Supplement: Supplementary file 2 — A. Search strategy for Ovid MEDLINE Epub Ahead of Print, In-Process & Other Non-Indexed Citations, Ovid MEDLINE Daily and Ovid MEDLINE. Data S2 B. Search strategy for EMBASE. Data S2 C. Search strategy for Scopus. Data S3 Exclusion of studies that had the same patient cohort. Data S4 Data of the included cohort study. Data S5 Quality assessment of included paper by Newcastle-Ottawa scale (NOS). Data S6 Adjusted odds ratios shown in the included studies. (DOCX 41 kb) [file 12876_2017_696_MOESM2_ESM.docx]

**Supplemental data 2A.** Search strategy for Ovid MEDLINE Epub Ahead of Print, In-Process & Other Non-Indexed Citations, Ovid MEDLINE Daily and Ovid MEDLINE

| **#** | **Searches** | **Results** |
| --- | --- | --- |
| 1 | (cholangiocarcinoma* or klatskin*).mp. or exp biliary tract neoplasms/ [mp=title, abstract, original title, name of substance word, subject heading word, keyword heading word, protocol supplementary concept word, rare disease supplementary concept word, unique identifier, synonyms] | 30312 |
| 2 | ((biliary or bile*) adj4 (cancer* or carcinoma*)).mp. [mp=title, abstract, original title, name of substance word, subject heading word, keyword heading word, protocol supplementary concept word, rare disease supplementary concept word, unique identifier, synonyms] | 6476 |
| 3 | 1 or 2 | 32186 |
| 4 | nafld.mp. or Non-alcoholic Fatty Liver Disease/ or nash.mp. | 10667 |
| 5 | ((nonalcohol* or "non alcohol*") adj2 (fatty or steatohepat* or liver)).mp. [mp=title, abstract, original title, name of substance word, subject heading word, keyword heading word, protocol supplementary concept word, rare disease supplementary concept word, unique identifier, synonyms] | 16088 |
| 6 | fatty liver/ | 19541 |
| 7 | 3 and (4 or 5 or 6) | 102 |
| 8 | 3 and risk factors/ | 1502 |
| 9 | (risk adj factor*).mp. [mp=title, abstract, original title, name of substance word, subject heading word, keyword heading word, protocol supplementary concept word, rare disease supplementary concept word, unique identifier, synonyms] | 931399 |
| 10 | 3 and 9 | 2126 |
| 11 | 7 or 8 or 10 | 2202 |
| 12 | 11 and (cohort* or prospective* or control* or retrospective* or trial* or "case control*").mp. [mp=title, abstract, original title, name of substance word, subject heading word, keyword heading word, protocol supplementary concept word, rare disease supplementary concept word, unique identifier, synonyms] | 1198 |
| 13 | 11 and incidence.mp. [mp=title, abstract, original title, name of substance word, subject heading word, keyword heading word, protocol supplementary concept word, rare disease supplementary concept word, unique identifier, synonyms] | 601 |
| 14 | 12 or 13 | 1450 |
| 15 | limit 14 to "all adult (19 plus years)" | 1018 |
| 16 | 14 and (adult* or men or women or population* or elder* or senior* or retired or older).mp. [mp=title, abstract, original title, name of substance word, subject heading word, keyword heading word, protocol supplementary concept word, rare disease supplementary concept word, unique identifier, synonyms] | 931 |
| 17 | 15 or 16 | 1141 |
| 18 | remove duplicates from 17 | 1067 |

**Supplemental data 2B.** Search strategy for EMBASE

| **#** | **Searches** | **Results** |
| --- | --- | --- |
| 1 | exp bile duct carcinoma/ | 20041 |
| 2 | cholangiocarcinoma*.mp. or exp bile duct cancer/ [mp=title, abstract, heading word, drug trade name, original title, device manufacturer, drug manufacturer, device trade name, keyword, floating subheading] | 24257 |
| 3 | 1 or 2 | 24257 |
| 4 | risk factor/ | 919120 |
| 5 | 3 and 4 | 1535 |
| 6 | fatty liver/ or exp nonalcoholic fatty liver/ | 49629 |
| 7 | (nash or nafl*).mp. [mp=title, abstract, heading word, drug trade name, original title, device manufacturer, drug manufacturer, device trade name, keyword, floating subheading] | 21637 |
| 8 | 6 and 7 | 18269 |
| 9 | ((nonalcohol* or "non alcohol*") adj2 (fatty or steatohepat* or liver or steato*)).mp. [mp=title, abstract, heading word, drug trade name, original title, device manufacturer, drug manufacturer, device trade name, keyword, floating subheading] | 30069 |
| 10 | 3 and 9 | 164 |
| 11 | 5 or 10 | 1651 |
| 12 | limit 11 to human | 1559 |
| 13 | exp case control study/ or exp case study/ or exp clinical trial/ or exp "clinical trial (topic)"/ or exp longitudinal study/ or exp major clinical study/ or exp prospective study/ or exp retrospective study/ | 4048847 |
| 14 | 12 and (13 or cohort*.mp. or "cross section*".mp.) [mp=title, abstract, heading word, drug trade name, original title, device manufacturer, drug manufacturer, device trade name, keyword, floating subheading] | 848 |
| 15 | remove duplicates from 14 | 804 |

**Supplemental data 2C.** Search strategy for Scopus

| **#** | **Searches** | **Results** |
| --- | --- | --- |
| 1 | TITLE-ABS-KEY((cholangiocarcinoma* OR klatskin* OR ((bile OR biliary) W/4 (carcinoma* OR cancer*))))AND TITLE-ABS-KEY((nafld OR nash OR ((nonalcohol* OR "non alcohol*") W/4 (liver OR fatty OR steato*)) OR "risk factor*"))) AND (incidence OR population* OR cohort* OR "case control*" OR retrospective* OR prospective* OR trial* OR outcome*) AND NOT (PMID(1* OR 2* OR 3* OR 4* OR 5* OR 6* OR 7* OR 8* OR 9*)) | 388 |

**Supplemental data 3.** Exclusion of studies that had the same patient cohort

Studies from Chaiteerakij et al.[^14^](#_ENREF_14) and Haung et al.[^15^](#_ENREF_15) were excluded because the patient cohort was a part of the cohort in studies of Choi et al.[^16^](#_ENREF_16) and Chang et al., respectively.[^17^](#_ENREF_17) As compared to the Choi et al study.[^16^](#_ENREF_16), Chaiteerakij et al.[^14^](#_ENREF_14) study had a smaller study population, with lower scores in quality assessment. Studies from Chang et al.[^17^](#_ENREF_17) and Haung et al.[^15^](#_ENREF_15) had the same scores for quality assessment. However, the controlled group in the Chang et al. study [^17^](#_ENREF_17) was defined more clearly. Additionally, the diagnosis of NAFLD was made by ICD-9, which was the same method to other studies included in this meta-analysis. In the Huang et al. study, ICD-O was used for the NAFLD diagnosis.[^15^](#_ENREF_15)

**Supplemental data 4.**

The only cohort study from Doycheva et al.,[^21^](#_ENREF_21) comprised 81 primary sclerosing cholangitis patients, found that 2 of 7 (28%) patients with NAFLD developed CCA, whereas 11 of 74 (15%) patients without NAFLD developed CCA. Consequently, the estimated relative risk was 1.72 (95%CI: 0.45-6.56), *P*=0.43.

**Supplemental data 5.** Quality assessment of included paper by Newcastle-Ottawa scale (NOS)

| Author, year | Quality assessment score | | | Total score (9) | Quality |
| --- | --- | --- | --- | --- | --- |
|  | Selection (4) | Comparability (2) | Outcome (3) |  |  |
| Welzel et al.[^10^](#_ENREF_10)  2007 | 3 | 2 | 1 | 6 | Fair |
| Zhou et al.[^20^](#_ENREF_20)  2009 | 3 | 2 | 3 | 8 | Good |
| Chang et al.[^17^](#_ENREF_17)  2013 | 2 | 2 | 1 | 5 | Fair |
| Lee et al.[^18^](#_ENREF_18)  2015 | 3 | 2 | 3 | 8 | Good |
| Kinoshita et al.[^9^](#_ENREF_9)  2016 | 2 | 1 | 2 | 5 | Fair |
| Choi et al.[^16^](#_ENREF_16)  2016 | 3 | 2 | 2 | 7 | Fair |
| Stepien et al.^19^ 2016 | 2 | 2 | 3 | 7 | Fair |

| Author, year | AOR (95% CI) | | Adjusted factors |
| --- | --- | --- | --- |
| Welzel et al.[^10^](#_ENREF_10)  2007 | iCCA  eCCA | 3.0 (1.2-7.3)  2.4 (0.9-6.5) | Age, gender, ethnicity, state buy-in status |
| Zhou et al.[^20^](#_ENREF_20)  2009 | iCCA | 1.52 (0.43-5.43) | HBV, cirrhosis, cholelithiasis, liver cyst, liver hemangioma, hepatic schistosomiasis, smoking, alcohol |
| Chang et al.[^17^](#_ENREF_17)  2013 | iCCA  eCCA | 2.4 (1.9-2.9)  2.0 (1.5-2.6) | DM, cholelithiasis, HBV, HCV, chronic pancreatitis, IBD |
| Kinoshita et al.[^9^](#_ENREF_9)  2016 | iCCA | 3.36 (1.15-10.20) | Age, obesity, albumin, GGP |
| Choi et al.[^16^](#_ENREF_16)  2016 | CCA  iCCA  distal CCA  perihilar CCA | 1.10 (0.82-1.48)  1.40 (0.94-2.09)  2.33 (0.97-5.59)  0.61 (0.36-1.04) | Age, gender, ethnicity, obesity, HTN, DM, CVA, CAD, PAD, AF, PSC, biliary tract disease, cirrhosis, HBV, HCV, IBD, smoking |

**Supplemental data 6.** Adjusted odds ratios shown in the included studies

AF: atrial fibrillation, AOR: adjusted odds ratio, CAD: coronary arterial disease, CCA: cholangiocarcinoma, CVA: cardiovascular accident, DM: diabetes mellitus, eCCA: extrahepatic cholangiocarcinoma, GGP: gamma-glutamyl transpeptidase, HBV: hepatitis B virus infection, HCV: hepatitis C virus infection, HTN: hypertension, IBD: inflammatory bowel disease, iCCA: intrahepatic cholangiocarcinoma, PAD: peripheral arterial disease
